# Tuberculosis in Brazil and cash transfer programs: A longitudinal database study of the effect of cash transfer on cure rates

**DOI:** 10.1371/journal.pone.0212617

**Published:** 2019-02-22

**Authors:** Barbara Reis-Santos, Priya Shete, Adelmo Bertolde, Carolina M. Sales, Mauro N. Sanchez, Denise Arakaki-Sanchez, Kleydson B. Andrade, M. Gabriela M. Gomes, Delia Boccia, Christian Lienhardt, Ethel L. Maciel

**Affiliations:** 1 Laboratory of Epidemiology of Federal University of Espírito Santo, Vitória/ES, Brazil; 2 Division of Pulmonary and Critical Care Medicine, University of California San Francisco, San Francisco, CA, United States of America; 3 Departamento de Estatística, Universidade Federal do Espírito Santo, Vitória/ES, Brazil; 4 Departamento de Saúde Coletiva, Universidade de Brasília: Asa Norte, Brasília/DF, Brazil; 5 Programa Nacional de Controle da Tuberculose—Ministério da Saúde, Brasília/DF, Brazil; 6 Liverpool School of Tropical Medicine, Liverpool, United Kingdom; 7 CIBIO-InBIO, Centro de Investigação em Biodiversidade e Recursos Genéticos, Universidade do Porto, Vairão, Portugal; 8 London School of Hygiene and Tropical Medicine: Keppel St., Bloomsbury, London, United Kingdom; 9 Global TB Programme, World Health Organisation, Geneva, Switzerland; 10 Unité Mixte Internationale TransVIHMI (UMI 233 IRD–U1175 INSERM—Université de Montpellier), Institut de Recherche pour le Développement, Montpellier, France; Médecins Sans Frontières (MSF), SOUTH AFRICA

## Abstract

**Introduction:**

Tuberculosis incidence is disproportionately high among people in poverty. Cash transfer programs have become an important strategy in Brazil fight inequalities as part of comprehensive poverty alleviation policies. This study was aimed at assessing the effect of being a beneficiary of a governmental cash transfer program on tuberculosis (TB) treatment cure rates.

**Methods:**

We conducted a longitudinal database study including people ≥18 years old with confirmed incident TB in Brazil in 2015. We treated missing data with multiple imputation. Poisson regression models with robust variance were carried out to assess the effect of TB determinants on cure rates. The average effect of being beneficiary of cash transfer was estimated by propensity-score matching.

**Results:**

In 2015, 25,084 women and men diagnosed as new tuberculosis case, of whom 1,714 (6.8%) were beneficiaries of a national cash transfer. Among the total population with pulmonary tuberculosis several determinants were associated with cure rates. However, among the cash transfer group, this association was vanished in males, blacks, region of residence, and people not deprived of their freedom and who smoke tobacco. The average treatment effect of cash transfers on TB cure rates, based on propensity score matching, found that being beneficiary of cash transfer improved TB cure rates by 8% [Coefficient 0.08 (95% confidence interval 0.06–0.11) in subjects with pulmonary TB].

**Conclusion:**

Our study suggests that, in Brazil, the effect of cash transfer on the outcome of TB treatment may be achieved by the indirect effect of other determinants. Also, these results suggest the direct effect of being beneficiary of cash transfer on improving TB cure rates.

## Introduction

Tuberculosis (TB) remains a major health concern and is the leading cause of infectious disease mortality worldwide [1]. In 2015, there were 10.4 million new TB cases and about 1.4 million of deaths, according to the World Health Organization (WHO) estimates. Despite some improvements, Brazil remains among the highest burden countries in the world with nearly 70,000 incident cases per year [1, 2].

The effect of poverty on TB incidence is unquestionable. The World Health Organization’s End TB Strategy emphasizes this relationship and supports the integration of social support policies in a comprehensive approach to TB elimination [3]. The Millennium Development Goals (MDGs) and, more recently, The Sustainable Development Goals (SDGs) have called upon governments to combat poverty and its consequences, including health conditions like TB, in order to enhance global social and economic development [2, 4]. For this reason, cash transfer programs have become an important strategy to fight inequalities in Brazil. The basic principle of cash transfer supposes that transfer of cash benefits directly affects poverty and poverty alleviation for individuals as well as acts indirectly to improve other non-economic outcomes. As such, cash transfers in Brazil have a place in a comprehensive national social protection policy [5].

Launched in 2003, the Bolsa Família Program (BFP) a national conditional cash transfer program that requires education and health conditionalities to be met by the household for the receipt of benefits, including school attendance, children’s immunizations, and pre- and post-natal care [5, 6]. The amount received is the sum of different Bolsa Família benefits, which vary according to family composition and income [5, 6]. In addition, the Continuous Cash Benefit (Benefício de Prestação Continuada–BPC) is a constitutional right, implemented since 1995, that involves a monthly unconditional cash transfer of a minimum wage (minimum amount of remuneration that an employer is required to pay wage earners for the work performed during a given period) targeted to people of any age with severe disabilities and to people older than 65 years old, who have family per capita income less than one-fourth of the minimum wage [6].

A systematic review showed that, in low and middle-income countries, effective social support, through income transfer mechanisms and comprehensive interventions, have beneficial influences on TB outcomes [7]. The effect of the Brazilian cash transfer programs, specifically, have been evaluated by several investigators. BFP is associated with improvements in children’s educational outcomes [8], parental participations in the labor force [9], childhood mortality [10], and leprosy incidence [11] among other social and health related outcomes. Also, Family Health Strategy and BFP had a positive impact on the TB mortality rate in Brazil [12]. A recent cohort study of people with TB showed that BFP has a direct effect on improving TB treatment outcomes [13], in agreement with a previous found from a longitudinal database study [14].

In order to understand the role of these governmental benefits on people with TB, since 2014, the Brazilian National Tuberculosis Program requires indication of the BFP beneficiary status on the TB notification forms. This study was aimed at assessing the effect of being beneficiary of a Brazilian governmental cash transfer program (BFP) on TB treatment cure rates.

## Methods

### Design and population

We investigated the association between TB determinants and the outcomes of TB treatment in people from Brazilian Notifiable Disease Information System (SINAN) through a longitudinal database study. We included people ≥18 years old with confirmed incident TB who started and finished their treatment in 2015. People with a previous TB treatment were excluded, as well as those from the state of São Paulo because, in 2015, this state did not incorporate the new set of SINAN variables including beneficiary status into their surveillance systems. SINAN is a public database for all notifiable diseases in the Brazilian Unified Healthcare System (SUS–Sistema Único de Saúde). The World Health Organization (WHO) uses SINAN data in estimating Brazilian TB incidence. These data also include socio-demographic and clinical characteristics, features of treatment and outcome for individuals diagnosed with TB [15, 16]. SINAN covers data since the 1990s and is reported to have a high degree of reliability [16, 17].

### Variables

Since 2014, in the beginning of treatment, people diagnosed with TB were asked about their governmental social programs benefits. A cash transfer beneficiary is considered any household member in a beneficiary family. These beneficiaries were divided into two groups based on classification within the SINAN database: a cash transfer group (people who answered as being beneficiaries of cash transfer program), and a non-cash transfer group (people who answered as not being beneficiaries of cash transfer program).

Concerning the outcomes of TB treatment, studied participants were prospectively followed through the treatment, from diagnosis to outcome, with TB outcome categorized as cure, dropout, death (from TB or from another cause), change of drug regimen, development of drug-resistant TB, and treatment failure. Cure is defined as treatment success which includes bacteriologically confirmed cure and completed treatment. The variable ‘treatment outcome’ was dichotomized in ‘cured’ and ‘not cured’ (including all other outcomes).

In order to explore SINAN covariates involved in the causal pathway to determining TB treatment outcomes, we also assessed the determinants proposed by the theoretical model of Maciel and Reis-Santos (2015) according to their vulnerability axes [16]. Individual vulnerability axis determinants were self-reported during medical evaluation and included: age (<40 years, 40–59 years, ≥60 years); sex (female, male); years of schooling (no schooling, 1–4 years, 5–8 years, >8 years); skin color/ethnicity (white, black, brown, yellow/indigenous); tobacco smoking and people with: disorder by alcohol consumption, disorder by illicit drugs use, diabetes, mental disorder, and HIV. Variables of social vulnerability axis were also self-reported and included: place of residence (geographic region: North, Northeast, Middle-West, Southeast, and South); characteristic of area of residence (urban, rural, peri-urban); and the belonging to increased risk populations (deprived of their freedom, homeless, healthcare workers, and immigrants). Clinical characteristics were also abstracted including the type of TB [pulmonary, extrapulmonary (EPTB), or pulmonary + EPTB]; and whether people were assigned to directly observed treatment (DOT) or not. In the Brazilian context, DOT is defined as the observation of tuberculosis medicines intake daily, from Monday to Friday, or three times a week, by a health care professional [18].

### Data analysis

#### Missing data

Missing data are a serious concern in epidemiologic studies, particularly those using secondary data. SINAN has reasonable completeness as previously reported, but missing data is a problem for some covariates [17,19]. S1 Table shows that most variables had more than 80% completeness, however among 25,084 subjects in our sample, only 5,993 (24%) had completed all information in the dataset. Incomplete datasets can be analyzed using multiple imputation through random draws from distributions inferred from observed data. Multivariate imputation by chained equations (MICE) is one of these recommended methods for multiple imputation in electronic health-record data [20, 21]. We treated missing data by using an algorithm based on random forest MICE called “missForest”. This algorithm predicts individual missing values more accurately rather than take random draws from a distribution and it can be used to impute continuous and/or categorical data including complex interactions and nonlinear relations [22]. Random forest is an extension of classification and regression trees that uses bootstrap aggregation of multiple regression trees to reduce the risk of overfitting. It combines the predictions from many trees to produce more accurate predictions [23]. We choose MICE random forest with 1,000 trees. Evidence has suggested that random forest MICE produces less biased parameter estimates and confidence interval with better coverage [23]. The imputation analysis was performed using the program R Project, version 3.3.3 (R Group, Vienna Austria).

#### Assessing the determinants of TB treatment outcome

Poisson regression models with robust variance were carried out to assess the effect of TB determinants on cure rates in the whole population and among the cash transfer group [24]. “Not cured” was defined as the reference category in both analyses. The TB determinants were included in the regression models, according to the following hierarchical levels: level 1 (sex, age, skin color, and schooling), level 2 (place of residence and characteristic of area of residence), level 3 (being deprived of their freedom, living in homeless, healthcare workers, and immigrants), level 4 (tobacco smoking, living with: alcohol use disorder, substance abuse disorder, diabetes, mental health disorder, and HIV), and level 5 (DOT or not). Thus, the effect of each determinant on the outcomes is interpreted as having been adjusted for all the variables that belong to the hierarchical levels above it (distal), as well as for the determinants’ effects that coexist on the same level [16]. Furthermore, this analysis was performed with participants with all type of TB and was replicated in a subsample of participants with pulmonary TB only. Since data were provided by a longitudinal database, the estimates were presented as risk ratios (RR) and 95% confidence intervals (95%CI).

#### Effect of cash transfer program on TB treatment outcome

People covered by governmental social programs of cash transfer must meet eligibility criteria, including low income and compliance with conditions related to education and health [25]. However, this governmental benefit is not universal; there are some people who meet these criteria but are not beneficiaries of national cash transfer programs. As SINAN did not incorporate information about eligibility to social programs, non-beneficiaries of the cash transfer program are a heterogeneous group. Therefore, the assessment of effect of being beneficiary of cash transfer program was performed by using a propensity score approach.

Propensity score indicates the probability of allocation to exposure group given the measured covariates [26]. The inclusion of people on BFP is not at random and, consequently, a single evaluation between beneficiaries and non-beneficiaries is not appropriate because the effect of BFP could be related to background characteristics, which may be different between groups. Propensity score matching estimation of the conditional probability of a person being a beneficiary, according to observed characteristics, and overcoming the selection limitations [26]. We estimated the propensity score of being beneficiary of cash transfer program using a logit model. The propensity score model included the following set of variables as predictors: schooling, area of residence, being healthcare worker, and presence of comorbidities (presence of one or more of the following comorbidities: tobacco smoking, alcohol use disorder, substance abuse disorder, diabetes, mental health disorder, and HIV). These variables were defined *a priori* based on our previous theoretical model to determine TB treatment outcomes using a direct acyclic graph (DAG) [13, 16].

We used Poisson regression models with robust variance to evaluate the direct effect of government cash transfer program on TB treatment outcomes (“not cured” was the category of reference). The first adjusted model included the set of confounding variables to the direct effect of cash transfer program on the outcomes: schooling, skin color, area of residence, region of residence, being healthcare worker, and comorbidities. The second model was adjusted by the propensity score (as continuous variable) and, additionally, by skin color and region of residence, which were not contained in the propensity score. Again, the Poisson regression models were replicated in a subsample including only people with pulmonary TB.

The average effect of being beneficiary of cash transfer program was estimated by propensity-score matching in a 1:1 ratio (we consider a pair of observations a match if the absolute difference in the propensity score was less than 0.05) [27, 28]. In this analysis, the outcomes were dichotomized as cured versus not cured and repeated for participants with pulmonary TB. The results were presented as coefficients, which are the difference between the effects for beneficiaries of cash transfer program compared to non-beneficiaries, and 95%CI. We also assessed whether assignment to DOT moderated the association of being a beneficiary of cash transfer program and cure rates of TB treatment.

#### Sensitivity analyses

In addition, all analyses mentioned above were replicated using a dataset which included only individuals without any missing SINAN data (so, not subject to imputation). The results are shown in the supplementary material.

Data were analyzed using Stata 14 (Stata Corp., College Station, USA).

### Ethics approval

The study was approved by the ethics committee of Centro de Ciências da Saúde from Universidade Federal do Espírito Santo under the number 242,826.

### Role of the funding source

There was no involvement of the funding source in study design, collection, analysis, and interpretation of data, writing, or in the decision to submit the paper for publication. All the authors had full access to the data in the study and had final responsibility for this publication.

## Results

**Fig 1:**
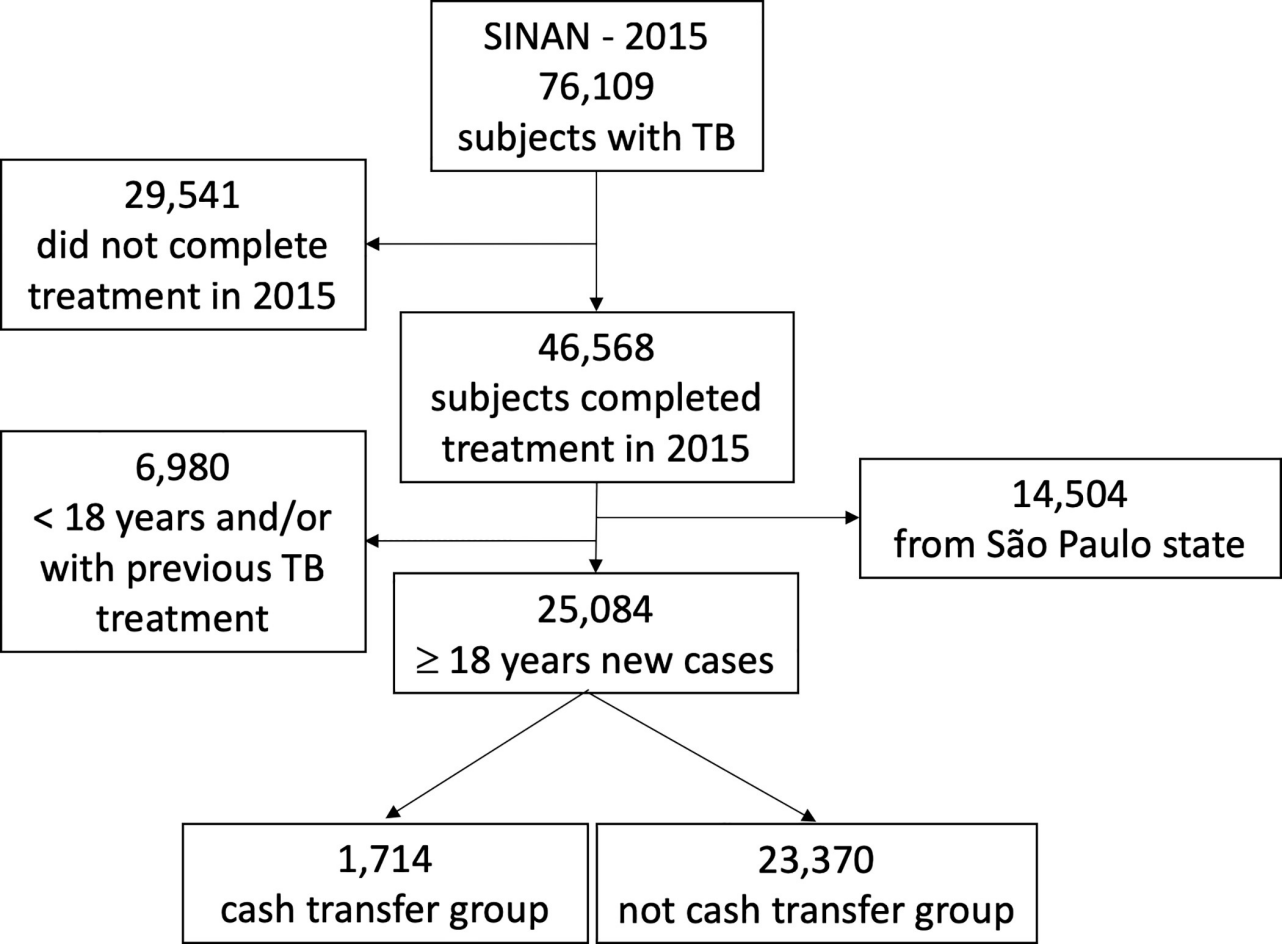
Flowchart of study sampling of people from the Brazilian Notifiable Disease Information System database (SINAN), 2015.

In 2015, there were 76,109 people with TB notified in SINAN, of which 46,568 finished their treatment in the same year. We excluded 14,504 people from São Paulo (without information about governmental benefits and 6,980 < 18 years old and/or with previous TB treatment (Fig 1). Thus, in the present analysis, we included 25,084 women and men aged ≥18 years old diagnosed as new TB case, of whom 1,714 (6.8%) were beneficiaries of governmental social program of cash transfer (cash transfer group).

### Sample description

Table 1 shows that 12,911 (51%) and 896 (52%) of whole population and cash transfer group were <40 years old, respectively. With respect to schooling, 2,405 (10%) of whole population and 328 (19%) of cash transfer group had no schooling and 7,360 (29%) of whole population and 598 (35%) of cash transfer group had one to four years of education. 7,405 (30%) of whole population, excluding all people from São Paulo state, live in the Southeast region while 763 (45%) of cash transfer group live in the Northeast region.

**Table 1:** Distribution of sociodemographic determinants of tuberculosis among people from the Brazilian Notifiable Disease Information System database (SINAN), 2015.

Determinants	Total population		Cash transfer group	
	n	%	n	%
**Sex**				
Women	8,330	33	942	55
Men	16,754	67	772	45
*Total*	*25*,*084*	*100*	*1*,*714*	*100*
**Age**				
<40 years	12,911	51	896	52
40–59 years	8,066	32	493	29
>60 years	4,107	16	325	19
*Total*	*25*,*084*	*100*	*1*,*714*	*100*
**Schooling**				
No schooling	2,405	10	328	19
1–4 years	7,360	29	598	35
5–8 years	7,467	30	495	29
>8 years	7,852	31	293	17
*Total*	*25*,*084*	*100*	*1*,*714*	*100*
**Skin color**				
White	7,832	31	309	18
Black	3,544	14	281	16
Brown	13,164	53	973	57
Yellow/Indigenous	544	2	151	9
*Total*	*25*,*084*	*100*	*1*,*714*	*100*
**Place of residence**				
North	3,739	15	280	16
Northeast	8,226	33	763	45
Southeast	7,405	30	283	17
South	4,113	16	214	12
Middle-West	1,601	6	174	10
*Total*	*25*,*084*	*100*	*1*,*714*	*100*
**Area of residence**				
Urban	22,511	90	1,297	76
Rural/peri-urban	2,573	10	417	24
*Total*	*25*,*084*	*100*	*1*,*714*	*100*
**People deprived of their freedom**				
No	23,185	92	1,624	95
Yes	1,899	8	90	5
*Total*	*25*,*084*	*100*	*1*,*714*	*100*
**Homeless**				
No	24,555	98	1,697	99
Yes	529	2	17	1
*Total*	*25*,*084*	*100*	*1*,*714*	*100*
**Healthcare worker**				
No	24,739	99	1,705	99
Yes	345	1	9	1
*Total*	*25*,*084*	*100*	*1*,*714*	*100*
**Immigrant**				
No	25,003	100	1,710	100
Yes	81	0	4	0
*Total*	*25*,*084*	*100*	*1*,*714*	*100*

Concerning behaviors and comorbidities (Table 2), among whole population and cash transfer group, respectively, 4,314 (17%) and 281 (16%) smoked tobacco, 4,314 (17%) and 214 (12%) lived with alcohol use disorder, 2,350 (9%) and 96 (6%) substance abuse disorder, 2,109 (8%) and 165 (10%) lived with diabetes, 609 (2%) and 53 (3%) lived with mental health disorder, and 2,815 (11%) and 128 (7%) lived with HIV.

**Table 2:** Distribution of clinical determinants of tuberculosis among people from the Brazilian Notifiable Disease Information System database (SINAN), 2015.

Determinants	Total population		Cash transfer group	
	n	%	n	%
**Tobacco smoking**				
No	20,770	83	1,433	84
Yes	4,314	17	281	16
*Total*	*25*,*084*	*100*	*1*,*714*	*100*
**Alcohol use disorder**				
No	20,770	83	1,500	88
Yes	4,314	17	214	12
*Total*	*25*,*084*	*100*	*1*,*714*	*100*
**Drug abuse disorder**				
No	22,734	91	1,618	94
Yes	2,350	9	96	6
*Total*	*25*,*084*	*100*	*1*,*714*	*100*
**Diabetes**				
No	22,975	92	1,549	90
Yes	2,109	8	165	10
*Total*	*25*,*084*	*100*	*1*,*714*	*100*
**Mental health disorder**				
No	24,475	98	1,661	97
Yes	609	2	53	3
*Total*	*25*,*084*	*100*	*1*,*714*	*100*
**HIV/AIDS**				
No	22,269	89	1,586	93
Yes	2,815	11	128	7
*Total*	*25*,*084*	*100*	*1*,*714*	*100*
**Clinical form**				
Pulmonary	21,163	84	1,504	88
EPTB or pulmonary + EPTB	3,921	16	210	12
*Total*	*25*,*084*	*100*	*1*,*714*	*100*
**DOT**				
No	12,643	50	578	34
Yes	12,441	50	1,136	66
*Total*	*25*,*084*	*100*	*1*,*714*	*100*
**Cash transfer program**				
No	23,370	93	0	0
Yes	1,714	7	1,714	100
*Total*	*25*,*084*	*100*	*1*,*714*	*100*
**Treatment outcome**				
Not cure	7,859	31	383	22
Cured	17,225	69	1,331	78
*Total*	*25*,*084*	*100*	*1*,*714*	*100*

DOT: directly observed therapy; EPTB: extrapulmonary tuberculosis; HIV/AIDS: human immunodeficiency virus/ acquired immunodeficiency syndrome; n: number of observations; TB: tuberculosis.

### Determinants of TB treatment outcome

The association of TB determinants and treatment outcome is showed in Table 3. Men demonstrated a lower probability of cure when compared with women (RR 0.90; 95%CI 0.88 to 0.91 in participants with all TB forms and RR 0.90; 95%CI 0.88 to 0.92 in participants with pulmonary TB). However, this difference vanished among men and women in the cash transfer group (RR 0.97; 95%CI 0.92 to 1.02 for participants with all TB forms and RR 0.98; 95%CI 0.93 to 1.03 for participants with pulmonary TB). When compared with people with no schooling, the risk ratio for cure was higher for those who studied > 8 years among total population (RR 1.19; 95%CI 1.15 to 1.23 to people with pulmonary TB), but was not seen among cash transfer group (RR 0.93; 95%CI 0.86 to 1.02 to people with pulmonary TB). Among the regions of Brazil, only people from the Northeast, when compared with those from Southeast, had higher cure rates (RR 1.12; 95%CI 1.02 to 1.21 in people with pulmonary TB).

**Table 3:** Adjusted* risk ratios of hierarchical Poisson regression models of the association of determinants of TB and treatment cure rates to 25,084 people from the Brazilian Notifiable Disease Information System database (SINAN), 2015.

Determinants	Risk ratios (95% confidence intervals) *			
	Total population		Cash transfer group	
	All TB forms	Pulmonary TB only	All TB forms	Pulmonary TB only
***Level 1***				
Sex				
Male	0.90 (0.88 to 0.91)	0.90 (0.88 to 0.92)	0.97 (0.92 to 1.02)	0.98 (0.93 to 1.03)
Age				
40–59 years	0.96 (0.95 to 0.98)	0.98 (0.96 to 1.00)	0.93 (0.87 to 0.98)	0.93 (0.87 to 0.99)
>60 years	0.81 (0.79 to 0.84)	0.82 (0.80 to 0.85)	0.75 (0.68 to 0.82)	0.73 (0.67 to 0.81)
Schooling				
1–4 years	1.00 (0.97 to 1.04)	1.01 (0.97 to 1.04)	0.96 (0.90 to 1.03)	0.94 (0.88 to 1.01)
5–8 years	0.91 (0.88 to 0.95)	0.93 (0.90 to 0.97)	0.87 (0.81 to 0.94)	0.87 (0.80 to 0.94)
>8 years	1.17 (1.13 to 1.21)	1.19 (1.15 to 1.23)	0.93 (0.85 to 1.01)	0.93 (0.86 to 1.02)
Skin color				
Black	0.94 (0.91 to 0.96)	0.94 (0.91 to 0.97)	0.95 (0.86 to 1.05)	0.98 (0.88 to 1.08)
Brown	0.98 (0.96 to 1.00)	0.99 (0.97 to 1.01)	1.05 (0.98 to 1.13)	1.06 (0.98 to 1.15)
Yellow/Indigenous	1.15 (1.10 to 1.20)	1.15 (1.10 to 1.21)	1.07 (0.97 to 1.18)	1.07 (0.97 to 1.19)
***Level 2***				
Region of residence				
North	1.11 (1.08 to 1.14)	1.14 (1.11 to 1.17)	1.05 (0.96 to 1.16)	1.10 (0.99 to 1.21)
Northeast	1.10 (1.08 to 1.13)	1.10 (1.08 to 1.13)	1.08 (0.99 to 1.17)	1.12 (1.02 to 1.21)
Middle-West	1.09 (1.06 to 1.13)	1.11 (1.07 to 1.15)	0.99 (0.87 to 1.12)	0.99 (0.86 to 1.13)
South	0.93 (0.90 to 0.96)	0.95 (0.92 to 0.98)	0.97 (0.86 to 1.08)	0.99 (0.88 to 1.12)
Area of residence				
Rural or peri-urban	1.15 (1.12 to 1.18)	1.15 (1.12 to 1.18)	1.14 (1.07 to 1.20)	1.12 (1.06 to 1.19)
***Level 3***				
Deprived of their freedom	1.14 (1.10 to 1.17)	1.14 (1.11 to 1.18)	1.12 (1.04 to 1.22)	1.09 (0.99 to 1.19)
Homeless	0.54 (0.48 to 0.60)	0.53 (0.47 to 0.60)	0.73 (0.48 to 1.11)	0.68 (0.42 to 1.10)
Healthcare worker	0.99 (0.93 to 1.05)	0.94 (0.88 to 1.01)	1.16 (0.86 to 1.58)	1.08 (0.65 to 1.77)
Immigrant	1.09 (0.94 to 1.27)	1.04 (0.87 to 1.24)	1.23 (0.97 to 1.56)	1.32 (0.99 to 1.75)
***Level 4***				
Tobacco smoking	0.93 (0.90 to 0.96)	0.93 (0.90 to 0.96)	0.89 (0.81 to 0.98)	0.91 (0.83 to 1.01)
Alcohol use disorder	0.85 (0.82 to 0.87)	0.84 (0.81 to 0.87)	0.82 (0.73 to 0.91)	0.81 (0.72 to 0.91)
Drug use disorder	0.76 (0.73 to 0.80)	0.77 (0.73 to 0.81)	0.83 (0.69 to 0.99)	0.81 (0.67 to 0.97)
Diabetes	1.01 (0.98 to 1.04)	1.01 (0.98 to 1.04)	0.97 (0.89 to 1.07)	0.98 (0.89 to 1.07)
Mental health disorder	0.98 (0.92 to 1.04)	0.97 (0.90 to 1.03)	1.04 (0.93 to 1.15)	1.07 (0.97 to 1.18)
HIV/AIDS	0.42 (0.40 to 0.44)	0.44 (0.41 to 0.47)	0.46 (0.36 to 0.58)	0.52 (0.41 to 0.66)
***Level 5***				
DOT	1.21 (1.19 to 1.23)	1.22 (1.20 to 1.25)	1.17 (1.10 to 1.24)	1.15 (1.08 to 1.22)

* Adjusted for the determinants that belong to the hierarchical levels above, as well as for the determinants’ effects on the same level.

DOT: directly observed therapy; EPTB: extrapulmonary tuberculosis; HIV/AIDS: human immunodeficiency virus/ acquired immunodeficiency syndrome; n: number of observations; TB: tuberculosis.

References: treatment outcome (cure), age (<40 years), schooling (no schooling), skin color (white), region of residence (Southeast), area of residence (urban), people deprived of their freedom (no), people living in homeless (no), healthcare worker (no), immigrant (no), and DOT (no).

Cure rates were inversely associated with homeless status (RR 0.54; 95%CI 0.48 to 0.60 to participants with all TB forms and RR 0.53; 95%CI 0.47 to 0.60 to participants with pulmonary TB) among total population. However, among the cash transfer group, this association was not found (RR 0.73; 95%CI 0.48 to 1.11 for participants with all TB forms and RR 0.68; 95%CI 0.42 to 1.10 for participants with pulmonary TB). Having, alcohol use disorder, drug use disorder, or living with HIV/AIDS were associated with decreased cure rates in the models analyzed.

People assigned to DOT were more likely to experience TB cure when compared with those not assigned to DOT in the total studied population (RR 1.21; 95%CI 1.19 to 1.23 in participants with all TB forms and RR 1.22; 95%CI 1.20 to 1.25 in participants with pulmonary TB). This effect was sustained among people in the cash transfer group, however there was an attenuation in the estimates of effect (RR 1.17; 95%CI 1.10 to 1.24 in participants with all TB forms and RR 1.15; 95%CI 1.08 to 1.22 in participants with pulmonary TB).

### Effect of cash transfer program on TB treatment outcome

Table 4 shows that 68% (15,894) of people without cash transfer benefit were cured, while 78% (1,331) of people with cash transfer benefit were cured of TB according to our outcome definitions.

**Table 4:** Distribution and estimates of the direct effect of being beneficiary of governmental cash transfer program on tuberculosis treatment outcomes among people from the Brazilian Notifiable Disease Information System database (SINAN), 2015.

Determinants	All TB forms			Pulmonary TB only		
	Treatment outcome					
	Not cured n (%)	Cured n (%)	Total n (%)	Not cured n (%)	Cured n (%)	Total n (%)
No cash transfer group	7,476 (32)	15,894 (68)	23,370 (100)	6,174 (31)	13,485 (69)	19,659 (100)
Cash transfer group	383 (22)	1,331 (78)	1,714 (100)	316 (21)	1,188 (79)	1,504 (100)
Total	7,859 (31)	17,225 (69)	25,084 (100)	6,490 (31)	14,673 (69)	21,163 (100)
**Total population**						
Model A^a^	RR 1.14 (95%CI, 1.11 to 1.17)			RR 1.15 (95%CI, 1.12 to 1.18)		
Model B^b^	RR 1.13 (95%CI, 1.10 to 1.16)			RR 1.14 (95%CI, 1.10 to 1.17)		
Model C^c^	RR 1.13 (95%CI, 1.10 to 1.16)			RR 1.14 (95%CI, 1.10 to 1.17)		
***Not assigned to DOT***						
Model A^a^	RR 1.11 (95%CI, 1.04 to 1.17)			RR 1.15 (95%CI, 1.08 to 1.21)		
Model B^b^	RR 1.13 (95%CI, 1.07 to 1.20)			RR 1.16 (95%CI, 1.10 to 1.23)		
Model C^c^	RR 1.12 (95%CI, 1.05 to 1.18)			RR 1.15 (95%CI, 1.09 to 1.23)		
***Assigned to DOT***						
Model A^a^	RR 1.10 (95%CI, 1.07 to 1.13)			RR 1.10 (95%CI, 1.07 to 1.13)		
Model B^b^	RR 1.08 (95%CI, 1.05 to 1.11)			RR 1.08 (95%CI, 1.05 to 1.11)		
Model C^c^	RR 1.09 (95%CI, 1.05 to 1.12)			RR 1.09 (95%CI, 1.05 to 1.12)		
***ATE***^***d***^ ***total population***	Coeff. 0.08 (95%IC, 0.05 to 0.10)			Coeff. 0.08 (95%IC, 0.06 to 0.11)		
***ATE***^***d***^ ***not assigned to DOT***	Coeff. 0.06 (95%IC, 0.02 to 0.10)			Coeff. 0.08 (95%IC, 0.04 to 0.13)		
***ATE***^***d***^ ***assigned to DOT***	Coeff. 0.06 (95%IC, 0.03 to 0.09)			Coeff. 0.06 (95%IC, 0.03 to 0.09)		

a: Model unadjusted.

b: Model adjusted by schooling, skin color, healthcare worker, area of residence, region of residence, and comorbidities.

c: Model adjusted by propensity score, skin color, and region of residence.

d: Average treatment effect of being beneficiary of governmental social program of cash transfer on cure.

Coeff.: difference of the effects for beneficiaries of cash transfer program and non-beneficiaries; DOT: directly observed therapy; n: number of observations; RR: risk ratio

Not Cured: reference category of outcome.

Controlling for confounding variables, there was a positive direct effect of being beneficiary of cash transfer program on cure rates of TB treatment in Poisson models of both studied groups, participants with all TB form and participants with pulmonary TB alone (Table 4). The risk ratio of cure among the cash transfer group was 1.13 (95%CI: 1.10 to 1.16) in both the model adjusted by confounding variables individually and the propensity scored model. DOT moderated the effect of this association [RR 1.16 (95%CI, 1.10 to 1.23) among participants with pulmonary TB not assigned to DOT and RR 1.08 (95%CI, 1.05 to 1.11) among participants with pulmonary TB assigned to DOT].

**Fig 2:**
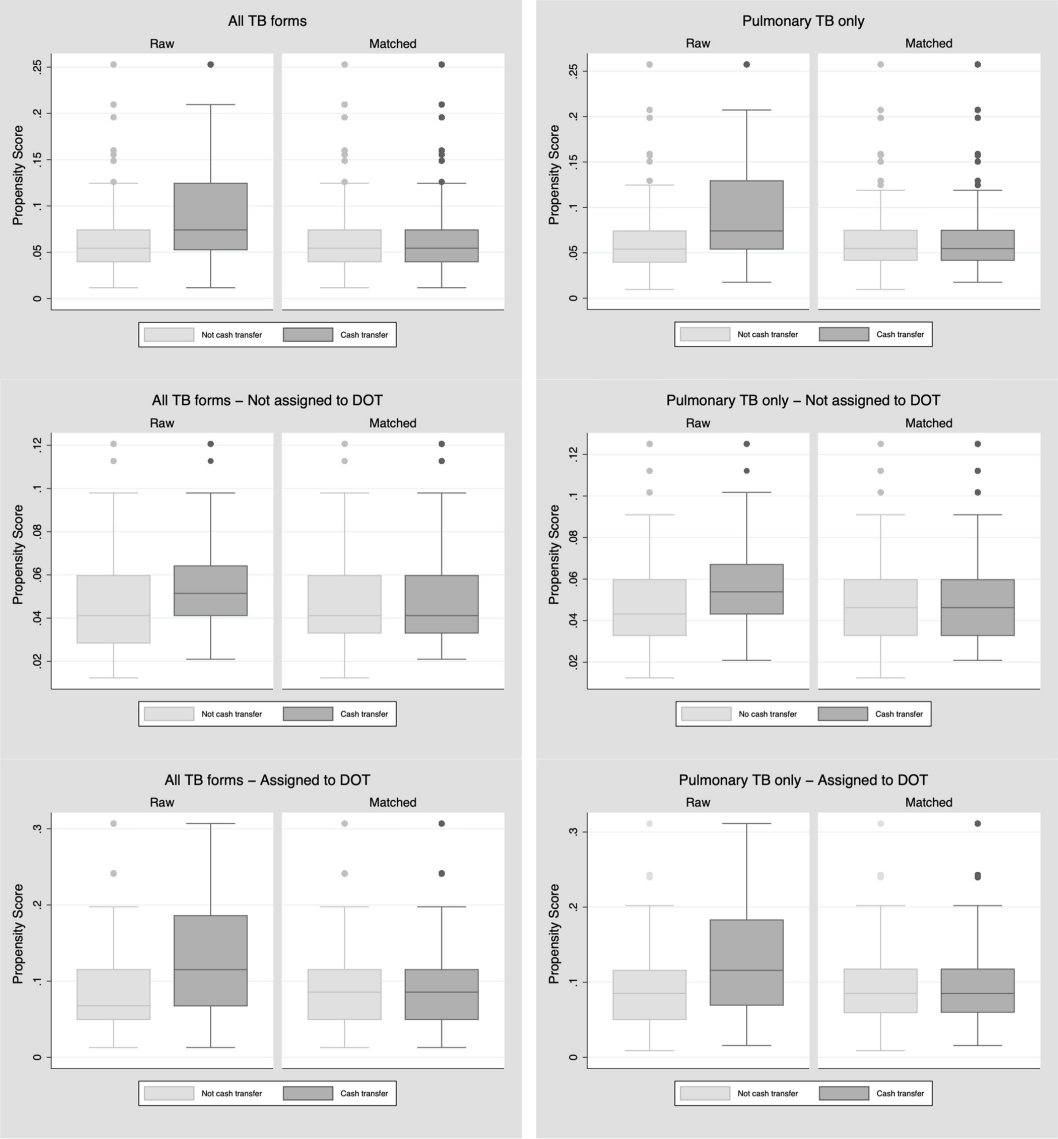
Balance box from propensity score matching of people from the Brazilian Notifiable Disease Information System database (SINAN-2015).

The average treatment effect of governmental program of cash transfer on TB cure rates, based on propensity score matching, found that being beneficiary of cash transfer improved cure rates by 8% [Coefficient 0.08 (95%CI: 0.05 to 0.10) in participants with TB of any type and Coefficient 0.08 (95%CI: 0.06 to 0.11) to participants with pulmonary TB only]. Also, the balance plots showed an improvement in the comparability between cash transfer groups after matching (Fig 2).

### Sensitivity analyses

All analyses were replicated with a dataset of people without missing information with did not undergo previous imputation, and they are available as supplementary material (S1, S2, S3 and S4 Tables and S1 Fig).

## Discussion

### Determinants of TB treatment outcome

In a Brazilian longitudinal database of people newly diagnosed with TB in 2015, we identified several determinants of TB treatment outcomes. Among the total population with pulmonary TB only, males, older subjects, those with 5–8 years of schooling, blacks, those living in the Southeast and South region and urban areas were less likely to be cured. Also were less likely to be cured those not deprived of their freedom, homeless, who smoke tobacco, living with alcohol use disorder, substance abuse or drug abuse disorder, HIV AIDS, and those assigned to DOT. However, among the cash transfer group, the association disappeared among males, blacks, region of residence, not deprived of their freedom, and who smoke tobacco products.

Previous assessment of SINAN database found similar associations among TB determinants [16]. New variables such as tobacco smoking, drug or substance use disorder, homeless, healthcare worker and immigrant status were recently included in the SINAN dataset based on evidence of high vulnerability among these people. Our results reinforce the role of these determinants in Brazil.

In general, studies evaluating the determinants of TB treatment outcome among beneficiaries of cash transfer programs are scarce. Torrens et. al. found, among people who were beneficiaries of BFP during TB treatment and after TB treatment, that cure was less likely in older subjects, illiterates, blacks, PLHIV/AIDS, those with unknown HIV status, and those not assigned to DOT [14].

In our present study, the effect of some determinants of TB treatment outcome in the total population was not maintained in the cash transfer group, suggesting that there might be an indirect effect of governmental social programs of cash transfer on TB treatment outcome in agreement with previous evidence [29]. Several mechanisms are pointed out to the potential impact of such intervention on TB treatment outcome and they include reducing financial barriers to accessing care and improving health behaviors [30].

### Effect of cash transfer program on TB treatment outcome

Our results suggest that there is an independent effect of benefit of cash transfer on improving cure rates of TB among people under TB treatment. This field of research is still incipient, but most of the studies evaluated the role of TB-specific initiatives of cash transfer on cure rates and they also found a positive association [13, 31].

With regard to TB-specific initiative, in Peru, people being treated for TB and their household contacts were randomly assigned to socioeconomic support (comprised conditional cash transfers up to $230 United States dollars). The socioeconomic support intervention was feasible and increased the proportion of household contacts of people being treated for TB who initiated TB preventive therapy; and the TB treatment success rate among people with TB [32].

To our knowledge, in Brazil, only two previous studies assessed the effect of a TB-sensitive program of cash transfer, the BFP, on TB treatment outcome [13, 14]. In the first published study, an analysis using secondary data, the authors found a positive association between BFP and TB cure and thus hypothesized an indirect effect of BFP through changes in behaviors and response to treatment [14]. The second study, based on a cohort of people with TB, estimated the average effect of being a BFP beneficiary on TB treatment outcomes and observed that the average effect for cure was 0.076 (IC 95% 0.037–0.116), for default was -0.070 (IC 95% - 0.105 - -0.036) and for death it was -0.002 (IC 95% - 0.021–0.017) [13]. Based on the same theoretical model to assess the direct effect of cash transfer, but using a secondary database, our study found similar results.

Catastrophic costs incurred when the total costs due to TB disease exceed 20% of the annual household income. Thus, social protection initiatives, TB-specific or not, appear to be a way to struggle against catastrophic costs, by alleviating the heavy economic burden of TB [33]. In Brazil, one study evaluated costs borne by TB-affected households and the proportion of households with catastrophic costs. The study evaluated the catastrophic costs in people with multidrug resistant (MDR) TB in Rio de Janeiro. The proportion ranged from 36% to 61% and confirmed that the social protection alleviates the catastrophic cost of these people [34].

The consistency between these results strengthens the evidence of a direct effect of BFP on TB treatment outcomes. However, given the nature of the data, we can not rule out the possibility that residual confounding may act in this association. Effect estimates reported by observational studies can be distorted by confounding. Generally, measurement error in a confounder and unmeasured confounders results in partial adjustment, biasing the association of interest in any direction [35]. The greatest challenges include the evaluations of social and behavioral determinants, since they involve complex causal chains, different dimensions, non-specific pathways and weak causal forces [36].

### Directly observed therapy

Evaluation based on the SINAN database with people notified with TB, in 2011, revealed that the DOT strategy targets vulnerable populations, as it is designed to do, and that it had an important impact on improving the TB cure rate in these populations [37]. Our results, with data from 2015, suggest an impact of DOT on TB treatment outcome even higher, suggesting that the role of DOT should not be disregarded in the struggle for TB control.

In agreement with previous findings [13, 14], our study showed that the direct effect of cash transfer was moderated by DOT, meaning that people not assigned to DOT had a higher direct effect of BFP. DOT improves the communication between health services and people with TB [37] and our hypothesis is that cash transfer benefit also work through this same mechanism. It is essential, therefore, to take into account that the protective effects of DOT and cash transfer benefit may combine synergistically in people covered by both. Further studies should assess the intermediate effect of DOT on the association between cash transfer benefit and TB treatment outcomes aiming at better understanding this relationship and developing the articulation between both strategies in Brazil.

### Strengths and limitations

The strengths of this study include the large longitudinal database with a countrywide sample, allowing a wide national representativeness. Moreover, we built the analytic models based on a rigorous theoretical model specific to Brazil and used multiple imputation techniques to deal with missing data. In spite of the known variation of missing data between states, and geographical regions, the reasons for such absence are related to health facilities characteristics, leading to a non-differential measurement error which was dealt with through multiple imputation. In the present study, we performed a sensitivity analysis to assess the likelihood of bias due to imputation by replicating our models with a dataset of people with completed information. Results with inclusive confidence intervals allow us to conclude that the main findings are consistent.

While governmental benefit was reported in the same way for beneficiaries and non-beneficiaries, the self-reported information may be a limitation by lowering accuracy and potential misclassification. As this error is likely to be non-differential, it would leave residual confounding. Heterogeneity among non-beneficiaries of cash transfer program also could be a limitation of our study. To overcome this, we used a propensity score approach to indicate the probability of allocation to exposure group and control for different variants of exposure. In addition, the assessments of social and behavioral aspects involve complex causal chains and contextual effects may also interact with these individuals’ characteristics altering the outcome determination [36]. Even performing an analysis based on a framework for causal thinking and adjusting to relevant social and behavioral determinants of health, we believe that residual confounding by these characteristics could be biasing our results.

DOT should be considered in SINAN as the observation of TB medicines taken, by a health professional, at least 3 times a week, in the attack phase, and at least 2 times a week, in the maintenance phase. However, there is no quality control to assure the consistency of this variable and DOT might not be correct classified in some health facilities. In spit of this limitation, is important to highlight that the initiative which led the health professional to assigned a people as under DOT contributed to better treatment outcomes.

### Conclusion

Our study suggests that, in Brazil, the effect of benefit of governmental cash transfer on the outcome of TB treatment may be achieved by other determinants, indirect effect. Also, there is suggestion of a direct effect of being beneficiary of cash transfer program on improving TB cure rates. Further studies should focus on elucidating the social and behavioral determinants which may play a role in the association of TB-sensitive interventions and the outcome of TB treatment, as well as on describing all causal paths involved in this association. Also, the effect of TB-specific interventions on the outcome of TB treatment as well as the role of strategies like DOT, with recognized effectiveness, in these association should be assessed.

## Supporting information

S1 Table Distribution of missing data among subjects from the Brazilian Notifiable Disease Information System database (SINAN), 2015. DOT: directly observed therapy; HIV/AIDS: human immunodeficiency virus/ acquired immunodeficiency syndrome; n: number of observations; TB: tuberculosis.
(PDF)

S2 Table Distribution of tuberculosis determinants of subjects with completed data on the Brazilian Notifiable Disease Information System database (SINAN), 2015. DOT: directly observed therapy; EPTB: extrapulmonary tuberculosis; HIV/AIDS: human immunodeficiency virus/ acquired immunodeficiency syndrome; n: number of observations; TB: tuberculosis.
(PDF)

S3 Table Adjusted* risk ratios of hierarchical Poisson regression models of the association of determinants of TB and treatment cure rates to subjects with completed data from the Brazilian Notifiable Disease Information System database (SINAN), 2015*. Adjusted for the determinants that belong to the hierarchical levels above, as well as for the determinants’ effects on the same level. DOT: directly observed therapy; EPTB: extrapulmonary tuberculosis; HIV/AIDS: human immunodeficiency virus/ acquired immunodeficiency syndrome; n: number of observations; TB: tuberculosis. References: treatment outcome (cure), age (<40 years), schooling (illiterate), skin color (white), region of residence (Southeast), area of residence (urban), prisoner (no), homeless (no), healthcare worker (no), immigrant (no), and DOT (no).
(PDF)

S4 Table Distribution and estimates of the direct effect of being beneficiary of governmental cash transfer program on tuberculosis treatment outcomes among subjects with completed data from the Brazilian Notifiable Disease Information System database (SINAN), 2015. a: Model unadjusted. b: Model adjusted by schooling, skin color, healthcare worker, area of residence, region of residence, and comorbidities. c: Model adjusted by propensity score, skin color, and region of residence. d: Average treatment effect of being beneficiary of governmental social program of cash transfer on cure. Coeff.: difference of the effects for beneficiaries of cash transfer program and non-beneficiaries; DOT: directly observed therapy; n: number of observations; RR: risk ratio. Not cured: reference category of outcome.
(PDF)

S1 Fig Balance box from propensity score matching of subjects with completed data from the Brazilian Notifiable Disease Information System database (SINAN-2015). (TIFF)
