# Supplementary material for: Tuberculosis in Brazil and cash transfer programs: A longitudinal database study of the effect of cash transfer on cure rates
Source: PLoS One. 2019 Feb 22;14(2):e0212617. doi: 10.1371/journal.pone.0212617 (PMC6386534; doi:10.1371/journal.pone.0212617)
Supplement: S1 Table — DOT: directly observed therapy; HIV/AIDS: human immunodeficiency virus/ acquired immunodeficiency syndrome; n: number of observations; TB: tuberculosis. (PDF) [file pone.0212617.s001.pdf]

S1 Table. Distribution of missing data among subjects from the Brazilian Notifiable Disease Information System database (SINAN), 2015.

Covariates	Complete data		Missing data	
	n	%	n	%
Age	25,073	99·96	11	0·04
Schooling	18,165	72·42	6,919	27·58
Skin color	23,345	93·07	1,739	6·93
Place of residence	25,084	100·00	0	0·00
Area of residence	24,297	96·86	787	3·14
Deprived of their freedom	20,696	82·51	4,388	17·49
Homeless	20,430	81·45	4,654	18·55
Healthcare worker	20,400	81·33	4,684	18·67
Immigrant	20,210	80·57	4,874	19,43
Tobacco smoking	21,371	85·20	3,713	14·80
Alcohol use disorder	22,466	89·56	2,618	10·44
Drug use disorder	21,210	84·56	3,874	15·44
Diabetes	22,282	88·83	2,802	11·17
Mental health disorder	22,203	88·51	2,881	11·48
HIV/AIDS	25,073	99·96	11	0·04
Treatment type	25,084	100·00	0	0·00
Clinical form	25,073	99·96	11	0·04
DOT	14,803	59·01	10,281	40·99
Cash transfer program	15,981	63·71	9,103	36·29
TB treatment outcome	18,939	75·50	6,145	24·50

DOT: directly observed therapy; HIV/AIDS: human immunodeficiency virus/ acquired immunodeficiency syndrome; n: number of observations; TB: tuberculosis.
