# Supplementary material for: Tuberculosis in Brazil and cash transfer programs: A longitudinal database study of the effect of cash transfer on cure rates
Source: PLoS One. 2019 Feb 22;14(2):e0212617. doi: 10.1371/journal.pone.0212617 (PMC6386534; doi:10.1371/journal.pone.0212617)
Supplement: S2 Table — DOT: directly observed therapy; EPTB: extrapulmonary tuberculosis; HIV/AIDS: human immunodeficiency virus/ acquired immunodeficiency syndrome; n: number of observations; TB: tuberculosis. (PDF) [file pone.0212617.s002.pdf]

S2 Table. Distribution of tuberculosis determinants of subjects with completed data on the Brazilian Notifiable Disease Information System database (SINAN), 2015.

Covariate	Total population		Cash transfer group	
	n	%	n	%
Age				
<40 years	2,907	50	207	45
40-59 years	1,916	33	150	33
>60 years	1,006	17	101	22
Schooling				
No schooling	407	7	64	14
1-4 years	1,607	28	162	35
5-8 years	1,872	67	140	31
>8 years	1,943	33	92	20
Skin color				
White	2,069	35	112	24
Black	751	13	70	15
Brown	2,902	50	246	54
Yellow	107	2	30	7
Place of residence				
North	703	12	76	16
Northeast	1,811	31	179	39
Southeast	1,377	24	69	15
South	1,572	27	94	21
Middle-West	366	6	40	9
Area of residence				
Urban	5,191	89	360	79
Rural/peri-urban	638	11	98	21
People deprived of their freedom	313	5	17	4
Homeless	118	2	4	1
Healthcare worker	99	2	2	0.5
Immigrant	25	0·5	0	0
Tobacco smoking	1,128	19	95	21
Alcohol use disorder	909	16	64	14
Drug abuse disorder	528	9	30	7
Diabetes	530	9	37	8
Mental health disorder	154	3	12	3
HIV/AIDS	536	9	43	9
Clinical form				
Pulmonary	4,962	85	401	88
EPTB or pulmonary + EPTB	867	15	57	12
DOT	2,894	50	281	61
Cash transfer program	458	8	458	100
Treatment outcome				
Cure	4,530	78	351	77
Dropout	723	12	49	11
Death from TB	246	4	26	6
Death from others causes	330	6	32	7
**Total**	**5,829**	**100**	**458**	**100**

DOT: directly observed therapy; EPTB: extrapulmonary tuberculosis; HIV/AIDS: human immunodeficiency virus/ acquired immunodeficiency syndrome; n: number of observations; TB: tuberculosis.
