# Supplementary material for: Tuberculosis in Brazil and cash transfer programs: A longitudinal database study of the effect of cash transfer on cure rates
Source: PLoS One. 2019 Feb 22;14(2):e0212617. doi: 10.1371/journal.pone.0212617 (PMC6386534; doi:10.1371/journal.pone.0212617)
Supplement: S3 Table — Adjusted for the determinants that belong to the hierarchical levels above, as well as for the determinants’ effects on the same level. DOT: directly observed therapy; EPTB: extrapulmonary tuberculosis; HIV/AIDS: human immunodeficiency virus/ acquired immunodeficiency syndrome; n: number of observations; TB: tuberculosis. References: treatment outcome (cure), age (<40 years), schooling (illiterate), skin color (white), region of residence (Southeast), area of residence (urban), prisoner (no), homeless (no), healthcare worker (no), immigrant (no), and DOT (no). (PDF) [file pone.0212617.s003.pdf]

S3 Table. Adjusted* risk ratios of hierarchical Poisson regression models of the association of determinants of TB and treatment cure rates to subjects with completed data from the Brazilian Notifiable Disease Information System database (SINAN), 2015.

Determinants	Risk ratios (95% confidence intervals)*			
	Total population		Cash transfer group	
	All TB forms	Pulmonary TB only	All TB forms	Pulmonary TB only
Level 1				
Sex				
Male	0·92 (0·89 to 0·95)	0·92 (0·89 to 0·95)	0·99 (0·90 to 1·10)	1·03 (0·93 to 1·15)
Age				
40-59 years	0·99 (0·96 to 1·02)	0·99 (0·96 to 1·03)	0·98 (0·88 to 1·10)	1·01 (0·91 to 1·14)
>60 years	0·93 (0·99 to 0·97)	0·93 (0·88 to 0·98)	0·77 (0·65 to 0·91)	0·75 (0·63 to 0·90)
Schooling				
1-4 years	1·05 (0·98 to 1·12)	1·02 (0·95 to 1·10)	0·97 (0·82 to 1·15)	0·97 (0·82 to 1·14)
5-8 years	1·00 (0·93 to 1·07)	0·98 (0·91 to 1·06)	0·86 (0·72 to 1·03)	0·85 (0·71 to 1·02)
>8 years	1·14 (1·06 to 1·22)	1·12 (1·04 to 1·20)	0·98 (0·82 to 1·18)	1·00 (0·83 to 1·20)
Skin color				
Black	0·97 (0·93 to 1·02)	0·98 (0·93 to 1·03)	0·87 (0·73 to 1·04)	0·92 (0·77 to 1·10)
Browns	1·01 (0·98 to 1·05)	1·01 (0·98 to 1·05)	0·94 (0·83 to 1·05)	0·99 (0·87 to 1·12)
Yellows	1·14 (1·05 to 1·24)	1·14 (1·04 to 1·24)	0·92 (0·72 to 1·16)	0·93 (0·72 to 1·19)
Level 2				
Region of residence				
North	1·03 (0·98 to 1·08)	1·06 (1·00 to 1·11)	1·13 (0·94 to 1·35)	1·23 (1·02 to 1·49)
Northeast	1·02 (0·98 to 1·05)	1·02 (0·98 to 1·06)	1·02 (0·86 to 1·21)	1·09 (0·90 to 1·31)
Middle-West	1·03 (0·97 to 1·09)	1·02 (0·95 to 1·09)	0·99 (0·77 to 1·29)	0·98 (0·73 to 1·32)
South	0·90 (0·87 to 0·95)	0·92 (0·88 to 0·97)	0·95 (0·78 to 1·15)	1·00 (0·81 to 1·24)
Area of residence				
Rural or peri-urban	1·06 (1·01 to 1·10)	1·06 (1·01 to 1·11)	1·11 (0·98 to 1·26)	1·08 (0·96 to 1·22)
Level 3				
Deprived of their freedom	1·06 (0·99 to 1·12)	1·07 (1·00 to 1·13)	1·09 (0·88 to 1·33)	1·06 (0·86 to 1·29)
Homeless	0·51 (0·41 to 0·65)	0·52 (0·40 to 0·66)	1·12 (0·65 to 1·93)	1·36 (1·09 to 1·69)
Healthcare worker	0·96 (0·87 to 1·06)	0·92 (0·81 to 1·04)	1·25 (1·06 to 1·47)	1
Immigrant	1·06 (0·87 to 1·30)	1·00 (0·78 to 1·28)	1	1
Level 4				
Tobacco smoking	0·95 (0·90 to 0·99)	0·96 (0·91 to 1·01)	0·88 (0·76 to 1·03)	0·88 (0·75 to 1·04)
Alcohol use disorder	0·92 (0·88 to 0·97)	0·93 (0·88 to 0·98)	0·92 (0·77 to 1·10)	0·92 (0·77 to 1·10)
Drug use disorder	0·85 (0·78 to 0·92)	0·85 (0·78 to 0·92)	1·02 (0·80 to 1·30)	1·06 (0·84 to 1·34)
Diabetes	0·99 (0·94 to 1·04)	0·99 (0·94 to 1·04)	0·86 (0·68 to 1·08)	0·89 (0·71 to 1·11)
Mental health disorder	0·97 (0·88 to 1·08)	0·98 (0·88 to 1·09)	0·80 (0·55 to 1·16)	0·92 (0·68 to 1·25)
HIV/AIDS	0·64 (0·59 to 0·71)	0·66 (0·60 to 0·73)	0·57 (0·42 to 0·78)	0·67 (0·47 to 0·94)
Level 5				
DOT	1·13 (1·09 to 1·16)	1·13 (1·10 to 1·17)	1·20 (1·07 to 1·34)	1·15 (1·03 to 1·30)

* Adjusted for the determinants that belong to the hierarchical levels above, as well as for the determinants' effects on the same level.

DOT: directly observed therapy; EPTB: extrapulmonary tuberculosis; HIV/AIDS: human immunodeficiency virus/ acquired immunodeficiency syndrome; n: number of observations; TB: tuberculosis.

References: treatment outcome (cure), age (<40 years), schooling (no schooling), skin color (white), region of residence (Southeast), area of residence (urban), people deprived of their freedom (no), people living in homeless (no), healthcare worker (no), immigrant (no), and DOT (no).
