# Supplementary material for: Tuberculosis in Brazil and cash transfer programs: A longitudinal database study of the effect of cash transfer on cure rates
Source: PLoS One. 2019 Feb 22;14(2):e0212617. doi: 10.1371/journal.pone.0212617 (PMC6386534; doi:10.1371/journal.pone.0212617)
Supplement: S4 Table — a: Model unadjusted. b: Model adjusted by schooling, skin color, healthcare worker, area of residence, region of residence, and comorbidities. c: Model adjusted by propensity score, skin color, and region of residence. d: Average treatment effect of being beneficiary of governmental social program of cash transfer on cure. Coeff.: difference of the effects for beneficiaries of cash transfer program and non-beneficiaries; DOT: directly observed therapy; n: number of observations; RR: risk ratio. Not cured: reference category of outcome. (PDF) [file pone.0212617.s004.pdf]

S4 Table. Distribution and estimates of the direct effect of being beneficiary of governmental cash transfer program on tuberculosis treatment outcomes among subjects with completed data from the Brazilian Notifiable Disease Information System database (SINAN), 2015.

**Determinants**	**All TB forms**			**Pulmonary TB only**		
	**Treatment outcome**					
	**Not cured n (%)**	**Cured n (%)**	**Total n (%)**	**Not cured n (%)**	**Cured n (%)**	**Total n (%)**
No cash transfer group	1,347 (24)	4,179 (76)	5,526 (100)	1,156 (25)	3,540 (75)	4,696 (100)
Cash transfer group	116 (25)	351 (75)	467 (100)	97 (24)	311 (76)	408 (100)
Total	1,463 (24)	4,530 (76)	5,993 (100)	1,253 (25)	3,851 (75)	5,104 (100)
**Total population**						
Model A[a]	RR 0·99 (95%CI, 0·94 to 1·05)			RR 1·01 (95%CI, 0·95 to 1·07)		
Model B[b]	RR 0·99 (95%CI, 0·94 to 1·05)			RR 1·00 (95%CI, 0·95 to 1·06)		
Model C[c]	RR 0·99 (95%CI, 0·94 to 1·05)			RR 1·01 (95%CI, 0·95 to 1·07)		
Not assigned to DOT						
Model A[a]	RR 0·94 (95%CI, 0·85 to 1·04)			RR 1·00 (95%CI, 0·90 to 1·11)		
Model B[b]	RR 0·95 (95%CI, 0·86 to 1·05)			RR 1·01 (95%CI, 0·91 to 1·12)		
Model C[c]	RR 0·95 (95%CI, 0·86 to 1·06)			RR 1·01 (95%CI, 0·91 to 1·12)		
Assigned to DOT						
Model A[a]	RR 1·00 (95%CI, 0·94 to 1·07)			RR 0·99 (95%CI, 0·93 to 1·06)		
Model B[b]	RR 1·00 (95%CI, 0·94 to 1·06)			RR 0·98 (95%CI, 0·92 to 1·05)		
Model C[c]	RR 1·00 (95%CI, 0·94 to 1·06)			RR 0·98 (95%CI, 0·92 to 1·05)		
ATE[d] total population	Coeff. -0·05 (95%IC, -0·05 to 0·03)			Coeff. 0·01 (95%IC, -0·04 to 0·05)		
ATE[d] not assigned to DOT	Coeff. -0·05 (95%IC, -0·12 to 0·02)			Coeff. -0·01 (95%IC, -0·09 to 0·07)		
ATE[d] assigned to DOT	Coeff. 0·01 (95%IC, -0·03 to 0·06)			Coeff. 0·01 (95%IC, -0·04 to 0·06)		

a: Model unadjusted.

b: Model adjusted by schooling, skin color, healthcare worker, area of residence, region of residence, and comorbidities.

c: Model adjusted by propensity score, skin color, and region of residence.

d: Average treatment effect of being beneficiary of governmental social program of cash transfer on cure.

Coeff.: difference of the effects for beneficiaries of cash transfer program and non-beneficiaries; DOT: directly observed therapy; n: number of observations; RR: risk ratio

Not cured: reference category of outcome
