# Supplementary figures and images for: Tuberculosis in Brazil and cash transfer programs: A longitudinal database study of the effect of cash transfer on cure rates
Source: PLoS One. 2019 Feb 22;14(2):e0212617. doi: 10.1371/journal.pone.0212617 (PMC6386534; doi:10.1371/journal.pone.0212617)

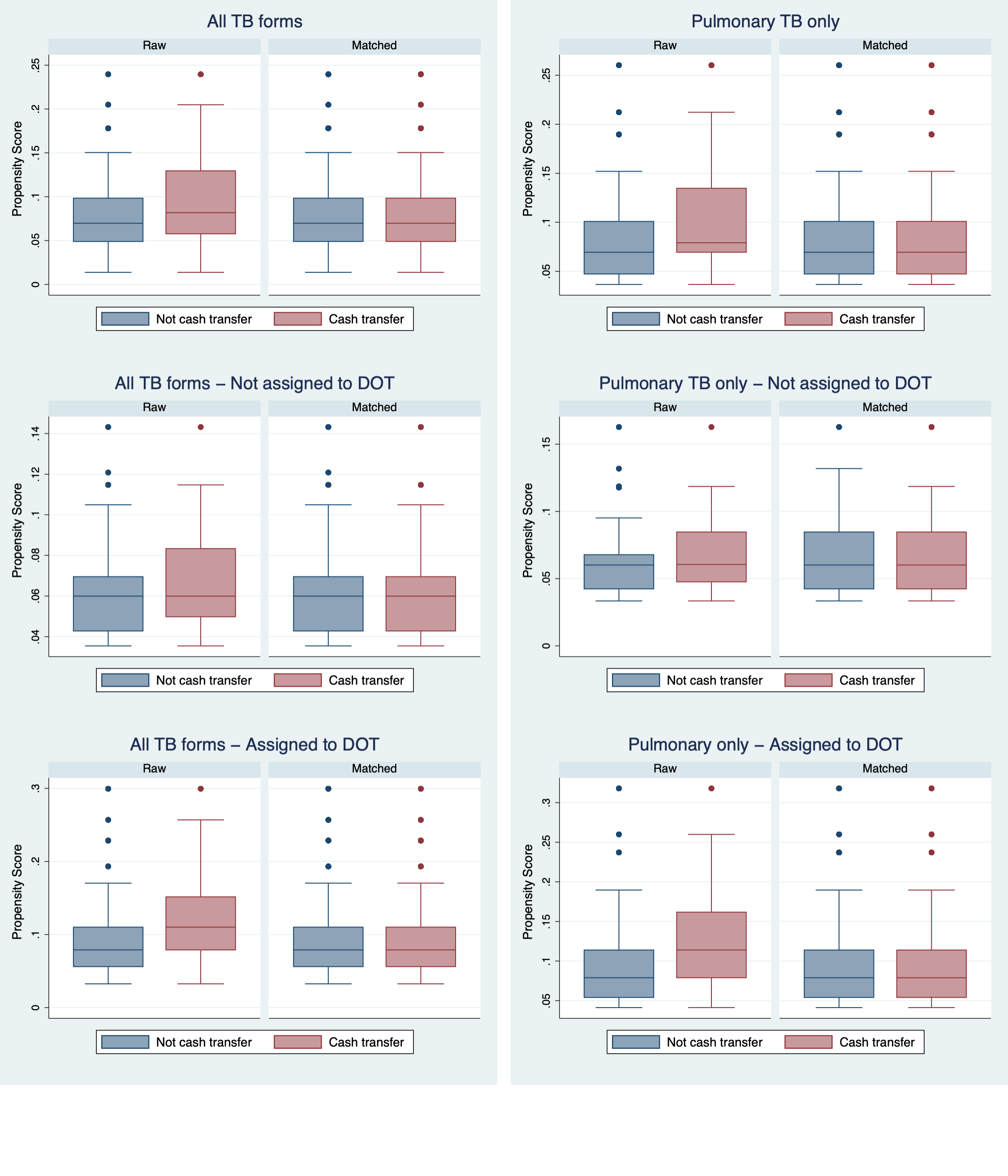

Supplement: S1 Fig — (TIFF) [file pone.0212617.s005.tiff]
